# Renoprotective Effects of Hypoxylonol C and F Isolated from *Hypoxylon* *truncatum* against Cisplatin-Induced Cytotoxicity in LLC-PK1 Cells

**DOI:** 10.3390/ijms19040948

**Published:** 2018-03-22

**Authors:** Buyng Su Hwang, Dahae Lee, Pilju Choi, Kyu Sun Kim, Seon-Jun Choi, Bong Geun Song, Taejung Kim, Ji Hoon Song, Ki Sung Kang, Jungyeob Ham

**Affiliations:** 1 Natural Products Research Institute, Korea Institute of Science and Technology, 679 Saimdang-ro, Gangneung 25451, Korea; hwang1531@nnibr.re.kr (B.S.H.); 090609@kist.re.kr (P.C.); 115042@kist.re.kr (K.S.K.); 117045@kist.re.kr (S.-J.C.); t16374@kist.re.kr (B.G.S.); kgsing@kist.re.kr (T.K.); 2 School of Pharmacy, Sungkyunkwan University, Suwon 16419, Korea; pjsldh@naver.com; 3 College of Korean Medicine, Gachon University, Seongnam 13120, Korea; 4 Department of Medicine, University of Ulsan College of Medicine, Seoul 05505, Korea; jhsong.john@gmail.com; 5 Division of Bio-Medical Science and Technology, University of Science and Technology, Daejeon 34113, Korea

**Keywords:** *Hypoxylon truncatum*, renoprotective effect, hypoxylonol

## Abstract

Although cisplatin is the standard platinum-based anticancer drug used to treat various solid tumors, it can cause damage in normal kidney cells. Protective strategies against cisplatin-induced nephrotoxicity are, therefore, clinically important and urgently required. To address this challenge, we investigated the renoprotective effects of *Hypoxylon* *truncatum*, a ball-shaped wood-rotting fungus. Chemical investigation of the active fraction from the methanol extract of *H.* *truncatum* resulted in the isolation and identification of the renoprotective compounds, hypoxylonol C and F, which ameliorated cisplatin-induced nephrotoxicity to approximately 80% of the control value at 5 μM. The mechanism of this effect was further investigated using hypoxylonol F, which showed a protective effect at the lowest concentration. Upregulated phosphorylation of p38, extracellular signal-regulated kinases, and c-Jun *N*-terminal kinases following cisplatin treatment were markedly decreased after pre-treatment with hypoxylonol F. In addition, the protein expression level of cleaved caspase-3 was significantly reduced after co-treatment with hypoxylonol F. These results show that blocking the mitogen-activated protein kinase signaling cascade plays a critical role in mediating the renoprotective effect of hypoxylonol F isolated from *H.* *truncatum* fruiting bodies.

## 1. Introduction

As the number of patients using anticancer drugs increases, there is growing concern about the side effects of anticancer drugs. The search for protective strategies against cisplatin (*cis*-diammine-dichloroplatinum II)-induced nephrotoxicity is of significant importance in cancer therapy. Cisplatin is an effective and widely used anticancer drug for many types of cancers including those of the breasts, ovaries, testes, and solid tumors of the head and neck [1,2]. However, cisplatin treatment is frequently discontinued because of its dose-dependent adverse effects on the normal kidney proximal tubules, where it decreases the glomerular filtration rate [1]. Recent studies showing that 25–40% of all cisplatin-treated patients suffer from nephrotoxicity highlight the clinical importance of protective strategies against cisplatin-induced nephrotoxicity [1,3,4].

Although various mechanisms of cisplatin-induced nephrotoxicity have been studied, it remains unclear how these mechanisms integrate to induce proximal tubular damage [5]. Cisplatin-induced proximal tubular damage is attributed to cellular damage caused by oxidative stress, DNA adducts, mitochondrial dysfunction, cell apoptosis, and inflammation [2,6,7]. Various types of cellular stress induced by cisplatin have been reported to activate mitogen-activated protein kinase (MAPK) pathways including c-JUN *N*-terminal kinase (JNK), extracellular signal-regulated kinase (ERK), and p38 kinase [2,8]. Specific inhibition of the MAPK superfamily, which is involved in directing cellular responses, reduces caspase-mediated apoptosis [2,9]. Recent studies have shown that inhibition of JNK, ERK, and p38 blocked caspase-3-mediated apoptosis in cisplatin-induced proximal tubular damage, although this mechanism remains unclear [4,10,11,12,13,14].

In recent decades, interest in the production of secondary metabolites from mushrooms has increased due to their various biological activities [10,11]. Among these, *Hypoxylon truncatum* (family Xylariaceae), an inedible ball-shaped wood-rotting fungus, was reported to contain unique benzo[*j*]fluoranthene compounds [12,13,14,15,16]. During our search for new bioactive secondary metabolites of Korean wild mushrooms, we collected the *H. truncatum* mushroom (Gangneung city, Korea) and isolated three major compounds from it: hypoxylonol C (**1**), hypoxylonol F (**2**), and 4,5,4′,5′-tetrahydroxy-1:1′-binaphthyl (BNT) (**3**), from a crude methanol extract. Although the hypoxylonol derivatives, as characteristic metabolites of *H. truncatum*, exhibited inhibitory activities against tumor angiogenesis [13,14], the various biological activities of its chemical constituents have not been researched fully. Especially, renoprotective effects against for anticancer drug-induced damage in kidney cells have not been reported so far to the best of our knowledge. Herein, we report our investigation of the renoprotective effects of hypoxylonol C (**1**), hypoxylonol F (**2**), and BNT (**3**). We hypothesized that hypoxylonol C (**1**), hypoxylonol F (**2**), and BNT (**3**) can activate MAPK-caspase-3 signaling to attenuate cisplatin-induced proximal tubular damage. We, therefore, investigated the role of MAPK-caspase-3 in renoprotection exerted by hypoxylonol C (**1**), hypoxylonol F (**2**), and BNT using an in vitro model. The goal of this study may aid the development of promising candidates for effective therapeutic intervention in cisplatin-induced kidney damage in cancer patients.

## 2. Results and Discussion

### 2.1. Protective Effect of H. truncatum Extract against Cisplatin-Induced LLC-PK1 Cell Death

Cisplatin-induced LLC-PK1 cell death is a major limitation for cancer patients undergoing cisplatin chemotherapy. Little research related to the biological activity of *H. truncatum* has been reported to date, and studies of the inhibitory activities of tumor angiogenesis are lacking [13,14]. Furthermore, the biological activities of the various chemical constituents of *H. truncatum* have not been fully studied. In the present study, we demonstrated for the first time the protective effects of the active constituents of *H. truncatum* against cisplatin-induced LLC-PK1 cell death, and the underlying mechanisms of these effects.

**Figure 1:**
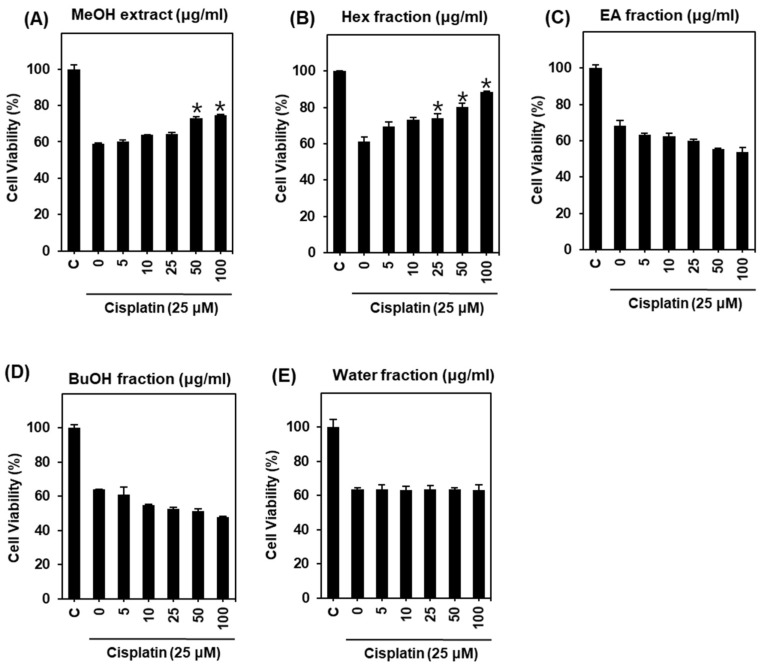
Comparison of protective effects of *H. truncatum* and its fractions in LLC-PK1 cells exposed to 25 μM of cisplatin for 24 h by MTT assay. Protective effect of (**A**) MeOH extract, (**B**) Hex fraction, (**C**) EA fraction, (**D**) BuOH fraction, and (**E**) water fraction in LLC-PK1 cells exposed to 25 μM of cisplatin for 24 h by MTT assay. Cell viability assays were done in triplicate for each assays and were repeated at least three times. * *p* < 0.05 compared with cisplatin-treated control.

To elucidate the protective effects of the *H. truncatum* extract, LLC-PK1 cells were treated with 25 μM cisplatin after pre-treatment with indicated concentrations of *H. truncatum* extract for 2 h. Cell viability was then measured using an MTT assay. As shown in Figure 1, cell viability was decreased to 61.04 ± 2.52% by treatment with 25 μM cisplatin for 24 h compared with untreated cells (Figure 1A). By contrast, *H. truncatum* MeOH extract showed a protective effect (Figure 1A). Cell viability of LLC-PK1 cells was significantly increased by pre-treatment with *H. truncatum* MeOH extract in a concentration-dependent manner. The maximum effect was observed at a concentration of 100 μg/mL (74.80 ± 0.18%) (Figure 1A). We next examined the protective effect of four fractions (water, *n*-BuOH, EA, and Hex) separated from the *H. truncatum* MeOH extract on cisplatin toxicity in LLC-PK1 cells. Only the Hex fraction showed a strong protective effect (Figure 1B–E). LLC-PK1 cell viability was significantly increased by pre-treatment with the Hex fraction in a concentration-dependent manner. The maximum effect was observed at a concentration of 100 μg/mL (88.68 ± 0.22%) (Figure 1B). Therefore, treatment with the Hex fraction appeared to confer protection against cell death induced by cisplatin. 

**Figure 2:**
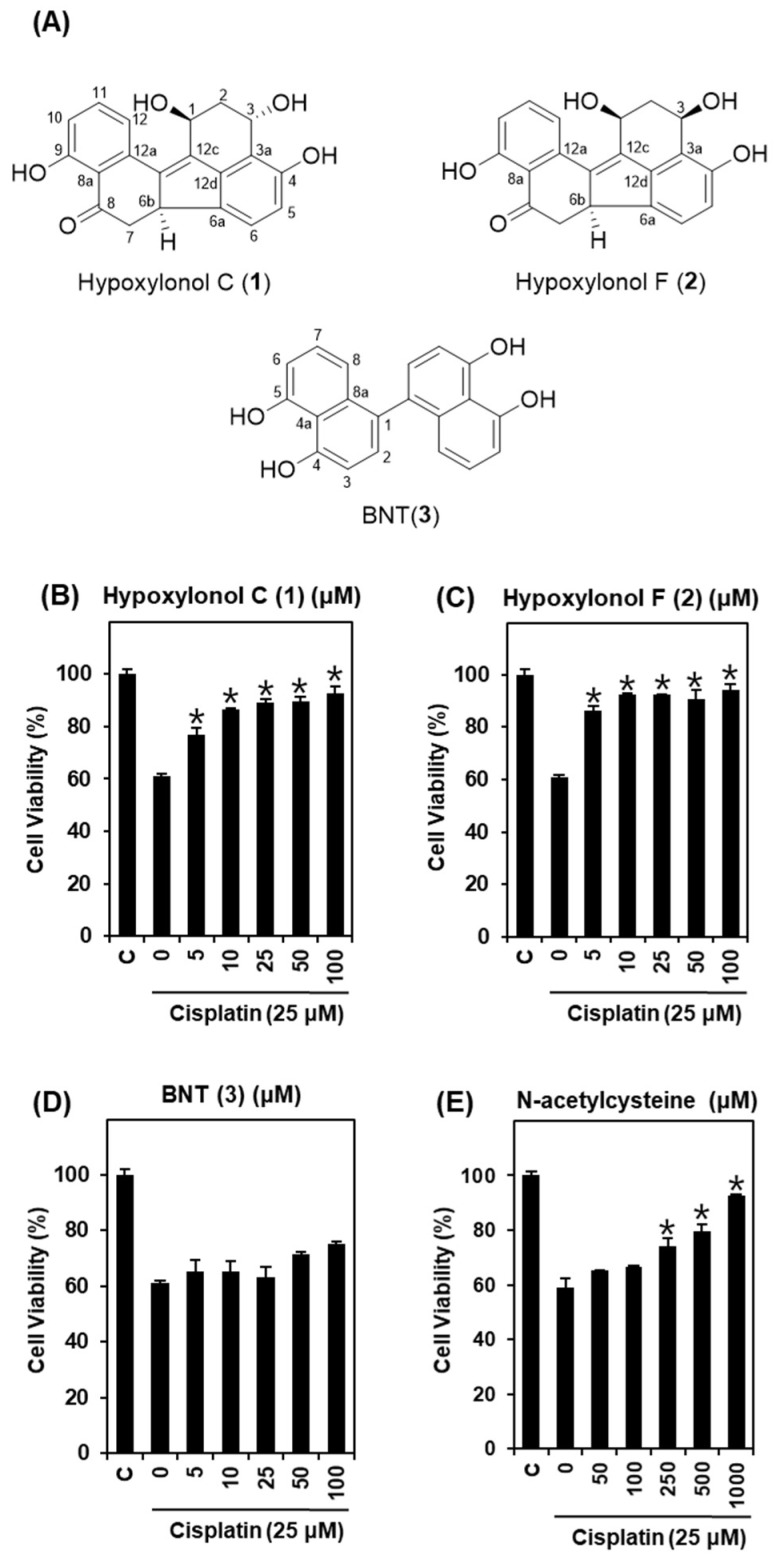
Comparison of protective effects of compounds **1**–**3** isolated from *H. truncatum* in LLC-PK1 cells exposed to 25 μM of cisplatin for 24 h by MTT assay. (**A**) Chemical structures of isolated compounds **1**–**3**. (**B**) Protective effects of hypoxylonol C (**1**) on cisplatin-induced cytotoxicity. (**C**) Protective effects of hypoxylonol F (**2**) on cisplatin-induced cytotoxicity. (**D**) Protective effects of BNT (**3**) on cisplatin-induced cytotoxicity. (**E**) Protective effects of *N*-acetylcysteine on cisplatin-induced cytotoxicity. Cell viability assays were done in triplicate for each assays and were repeated at least three times. * *p* < 0.05 compared with cisplatin-treated control.

Based on this result, we investigated the Hex fraction for renoprotective compounds using column chromatography and HPLC purification and identified hypoxylonol C (**1**), hypoxylonol F (**2**), and BNT (**3**) (Figure 2A). To elucidate the protective effect of these compounds, LLC-PK1 cells were treated with 25 μM cisplatin after pre-treatment with indicated concentrations of the three compounds for 2 h. Cell viability was then measured by MTT assay. As shown in Figure 2, cell viability decreased to 60.96 ± 0.84% following treatment with 25 μM cisplatin for 24 h compared with untreated cells. By contrast, hypoxylonol C and F at 5 μM ameliorated cisplatin-induced nephrotoxicity to approximately 80% of the control value. Among the three compounds, hypoxylonol F (**2**) in particular showed the strongest protective effect, even at a low concentration (Figure 2B–D). LLC-PK1 cell viability was significantly increased by pre-treatment with hypoxylonol F (**2**) in a concentration-dependent manner, with the maximum effect observed at a concentration of 5 μM (86.46 ± 1.72%) (Figure 2C). The protection effect of hypoxylonol F was superior to the effect of the positive control compound (*N*-acetylcysteine) at a concentration of 500 μM (79.38 ± 2.72%) (Figure 2E). *N*-acetylcysteine is known to prevent cisplatin-induced apoptosis through the regulation of myc proto-oncogene protein [17] and suppressed intracellular ROS [18]. In this study, a considerable renoprotective effect was observed for hypoxylonol F (**2**) at 5 and 10 μM, a lower concentration than for the other compound and *N*-acetylcysteine. To the best of our knowledge, this study is the first to demonstrate that hypoxylonol F from *H. truncatum* mitigates cisplatin-induced toxicity in LLC-PK1 cells. Therefore, further mechanistic studies were subsequently carried out using hypoxylonol F (**2**).

### 2.2. Effects of Hypoxylonol F (**2**) on Cisplatin-Induced Apoptosis in LLC-PK1 Cells

**Figure 3:**
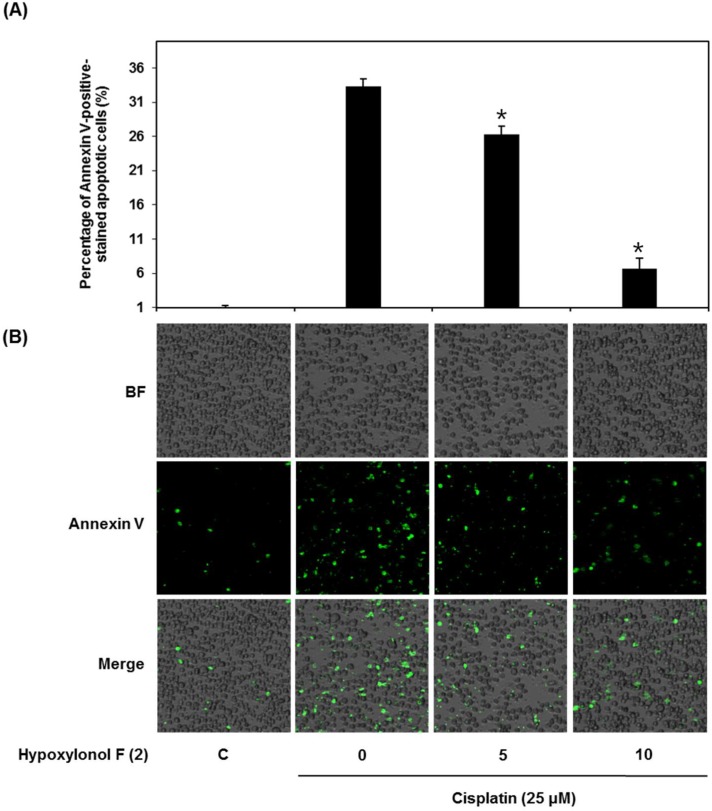
Effect of hypoxylonol F (**2**) on apoptosis in LLC-PK1 cells exposed to 25 μM of cisplatin for 24 h by image-based cytometric assay. (**A**) Percentage of annexin V-positive-stained apoptotic cells. (**B**) Representative images for apoptosis detection. BF (bright field): cell morphology under light microscope. Image-based cytometric assays were done in triplicate and were repeated at least two times. * *p* < 0.05 compared with cisplatin-treated control.

Cells were exposed to 25 μM cisplatin in the presence or absence of hypoxylonol F (**2**) and stained with annexin V conjugated with Alexa Fluor 488. As shown in Figure 3, the percentage of annexin V-positive cells, indicating apoptosis, was significantly increased to 33.33 ± 1.15% following treatment with 25 μM cisplatin, but was decreased to 26.33 ± 1.15% and 6.66 ± 1.52% by 50 μM and 100 μM of compound **2**, respectively (Figure 3A,B).

The underlying mechanisms for cisplatin-induced apoptotic proximal tubule cell death have been reported previously. Caspases are cysteine proteases, and play an essential role in the execution of the apoptosis pathway (death receptor-mediated extrinsic and mitochondria-mediated intrinsic pathway) [19,20]. Several studies have shown that caspase-3 is the downstream executioner responsible for the cleavage of various physiological substrates including DNA repair protein, which can be activated by DNA strand breakage [9,21]. Cisplatin causes DNA strand breakage, thus blocking replication and transcription, which may ultimately lead to cell cycle arrest and apoptosis [22].

Cisplatin induces apoptotic proximal tubule cell death by enhancing caspase-3 activation [9], which is preceded by the activation of JNK, ERK, and p38 [8,9]. In proximal tubular cell lines including LLC-PK1 [23], OK [24], HK-2 [23], TKPTS [4], and primary cultures of renal proximal tubular cells [25], inhibition of JNK, ERK, and p38 enhanced cell viability by inhibiting apoptosis after cisplatin treatment. Thus, the prevention of cisplatin-induced apoptotic cell death in renal cells is beneficial in reducing the side effects of cisplatin. In addition, JNK, ERK, and p38 pathways appear to be potential targets for cisplatin-induced apoptosis in LLC-PK1 cells. Therefore, we further explored whether hypoxylonol F (**2**) could decreased cisplatin-induced apoptosis in LLC-PK1 cells.

**Figure 4:**
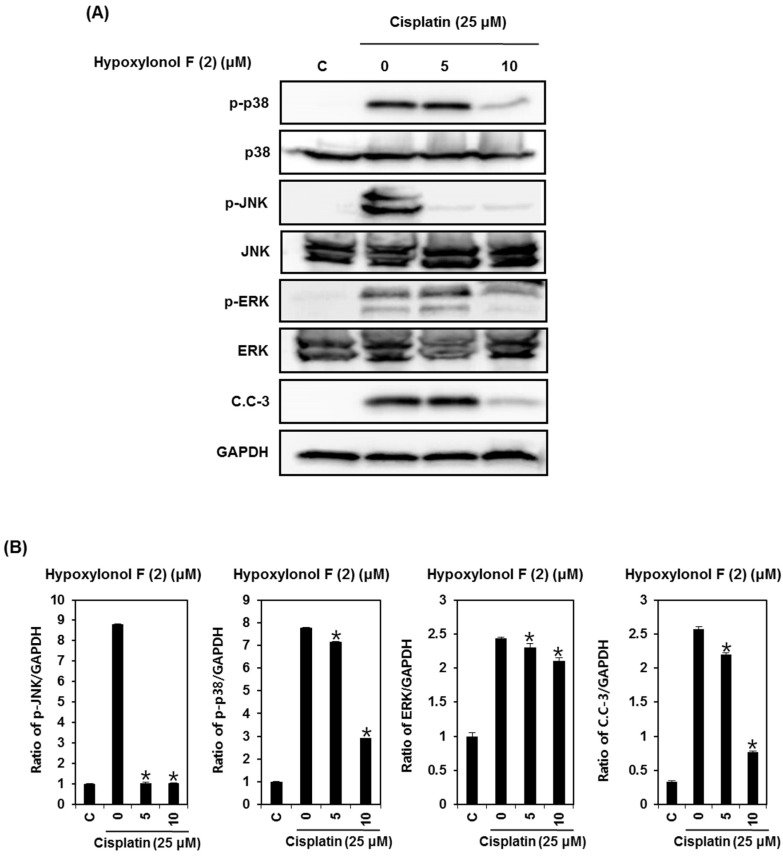
Effect of hypoxylonol F (**2**) on the expression of apoptosis-related proteins in LLC-PK1 cells exposed to 25 μM of cisplatin for 24 h. (**A**) Expression of proteins in the MAPK-caspase-3 pathway. (**B**) Each bar graph shows the densitometric quantification of western blotting bands. Western blot assays were repeated at least three times. * *p* < 0.05 compared with cisplatin-treated control.

To elucidate the underlying molecular mechanism of hypoxylonol F (**2**)-mediated protection against cisplatin toxicity, LLC-PK1 cells were exposed to 25 μM cisplatin for 24 h with or without 50 μM or 100 μM of hypoxylonol F (**2**), followed by Western blotting analysis to evaluate MAPK phosphorylation and caspase-3 cleavage. As shown in Figure 4, p38, JNK, and ERK phosphorylation was increased by 25 μM cisplatin, whereas it was markedly decreased by treatment with 5 μM and 10 μM hypoxylonol F (**2**) (Figure 4A). Quantitative analysis showed that p38, JNK, and ERK phosphorylation was significantly decreased in the presence of hypoxylonol F (**2**) compared with the cisplatin-treated group (Figure 4B). In addition, caspase-3 cleavage was significantly increased by cisplatin treatment, whereas it was significantly decreased in the presence of hypoxylonol F (**2**) (Figure 4A,B). In agreement with previous studies, these results indicate that the most critical signaling pathway in the protective effect of hypoxylonol F (**2**) against cisplatin-induced toxicity in LLC-PK1 cells is the inhibition of MAPK phosphorylation and cleavage of caspase-3.

## 3. Materials and Methods

### 3.1. General Experimental Procedures

Nuclear magnetic resonance (NMR) spectra were recorded on a Bruker AVACE III 400 NMR spectrometer (Bruker, Rheinstetten, Germany) operating at 400 MHz (^1^H) and 100 MHz (^13^C), with chemical shifts given in ppm (δ). Preparative high performance liquid chromatography (HPLC) was performed using a Gilson 306 pump (Gilson, Middleton, WI, USA), while HPLC analysis used a Shimadzu Nexera X2 HPLC system (Shimadzu, Kyoto, Japan) equipped with a UV detector (Shimadzu, SPD-M20A). Low-resolution electrospray ionization mass spectrometry (ESI-MS) data were measured using a Shimadzu LCMS-2020 system.

### 3.2. Mushroom Material

A *H. truncatum* specimen was collected in Yeongok-myeon, Gangneung city, Korea, in September 2016 and identified by Dr. Soon-Ja Seok at the National Academy of Agricultural Science, Rural Development Administration. A voucher specimen (MCO-NP-I-0026) was deposited at the Library of Natural Products Research Institute, Korea Institute of Science and Technology.

### 3.3. Extraction and Isolation

Dried and powdered *H. truncatum* (50 g) was extracted successively with MeOH (2 × 1 L) at room temperature and filtered. The filtrate was evaporated under a vacuum to obtain a MeOH extract. The crude extract (6.3 g) was used for the bioassay-guided fractionation and isolation of compounds. The methanolic extract was re-suspended in water and partitioned successively with hexane (Hx), ethyl acetate (EtOAc), and normal butanol (*n*-BuOH). The Hx fraction (1.2 g) was separated by preparative HPLC using a MeOH/H_2_O mobile phase (0–60 min: 50:50 to 100:0; *v*/*v*) and an ODS column (Phenomenex C18, 250 × 21.2 mm, 10 µm) at a flow rate of 8 mL/min to yield three fractions (A, B, and C). Purification of A, B, and C was performed by semi-preparative HPLC using a CH_3_CN/H_2_O mobile phase (0–60 min: 30:70 to 80:20) and an ODS column (Phenomenex Gemini C6 Phenyl, 250 × 10 mm, 5 µm) at a flow rate of 4 mL/min to afford compounds **1** (158 mg), **2** (67 mg), and **3** (119 mg) (Figure 2A). The chemical structures of these compounds were determined by comparison of their spectroscopic data (Figures S1–S15) with those of previously reported reference compounds [13].

**Hypoxylonol C (1).** Yellow amorphous powder. ^1^H-NMR (400 MHz, acetone-*d*_6_) δ 12.61 (s, 1H, OH-9), 8.65 (s, 1H, OH-4), 7.56 (t, 1H, *J* = 8.0 Hz, H-11), 7.49 (dd, 1H, *J* = 8.0, 1.2 Hz, H-12), 7.28 (d, 1H, *J* = 8.0 Hz, H-6), 6.84 (dd, 1H, *J* = 8.0, 1.2 Hz, H-10), 6.68 (d, 1H, *J* = 8.0 Hz, H-5), 5.59 (dd, 1H, *J* = 8.5, 4.2 Hz, H-1), 5.48 (m, 1H, H-3), 5.14 (brd, 1H, OH-3), 4.28 (s, 1H, OH-1), 4.11 (dd, 1H, *J* = 13.8, 5.5 Hz, H-6b), 3.38 (dd, 1H, *J* = 16.5, 5.6 Hz, H-7), 2.47 (dt, 1H, *J* = 13.0, 4.3 Hz, H-2), 2.31 (dd, 1H, *J* = 16.4, 14.0 Hz, H-7), 2.14 (ddd, 1H, *J* = 13.0, 8.5, 3.2 Hz, H-2); ^13^C-NMR (100 MHz, acetone-*d*_6_) δ 206.2 (C-8), 163.8 (C-9), 155.9 (C-4), 144.3 (C-12d), 139.6 (C-12a), 138.3 (C-12c), 137.9 (C-12b), 137.7 (C-11), 136.6 (C-6a), 123.7 (C-6), 120.8 (C-3a), 119.0 (C-12), 117.2 (C-10), 115.7 (C-8a), 114.4 (C-5), 65.5 (C-3), 62.8 (C-1), 49.9 (C-6b), 43.7 (C-7), 42.4 (C-2); HRFABMS *m*/*z* 335.0925 [M − H]^−^ (calcd. for C_20_H_15_O_5_, 335.0919).

**Hypoxylonol F (2).** Yellow amorphous powder. ^1^H-NMR (400 MHz, acetone-*d*_6_) δ 12.60 (s, 1H, OH-9), 7.53 (t, 1H, *J* = 8.0 Hz, H-11), 7.48 (dd, 1H, *J* = 8.0, 1.2 Hz, H-12), 7.29 (d, 1H, *J* = 8.0 Hz, H-6), 6.82 (dd, 1H, *J* = 8.0, 1.2 Hz, H-10), 6.69 (d, 1H, *J* = 8.0 Hz, H-5), 5.38 (d, 1H, *J* = 8.5 Hz, H-1), 5.11 (d, 1H, *J* = 8.5 Hz, H-3), 4.09 (dddd, 1H, *J* = 13.8, 5.4, 23.9, 0.7 Hz, H-6b), 3.39 (dd, 1H, *J* = 16.4, 5.5 Hz, H-7), 2.47 (dt, 1H, *J* = 12.6, 4.3 Hz, H-2), 2.34 (dd, 1H, *J* = 16.3, 13.8 Hz, H-7), 2.24 (dt, 1H, *J* = 12.6, 8.5 Hz, H-2); ^13^C-NMR (100 MHz, acetone-*d*_6_) δ 206.3 (C-8), 163.6 (C-9), 155.7 (C-4), 144.2 (C-12d), 139.4 (C-12a), 139.0 (C-12c), 137.5 (C-12b), 137.1 (C-11), 136.6 (C-6a), 123.8 (C-6), 121.0 (C-3a), 120.9 (C-12), 117.0 (C-10), 115.8 (C-8a), 114.6 (C-5), 67.0 (C-3), 65.2 (C-1), 50.1 (C-6b), 43.7 (C-7), 43.3 (C-2); HRFABMS *m*/*z* 337.1074 [M + H]^+^ (calcd. for C_20_H_17_O_5_, 337.1076).

**4,5,4′,5′-Tetrahydroxy-1:1′-binaphthyl, BNT (3).** Yellow amorphous powder. ^1^H-NMR (400 MHz, acetone-*d*_6_) δ 11.04 (br d, 4H, OH-4, 5), 7.16 (d, 2H, *J* = 8.0 Hz, H-7), 7.07 (t, 2H, *J* = 8.0 Hz, H-2), 6.83 (d, 2H, *J* = 8.0 Hz, H-3), 6.72 (dd, 2H, *J* = 8.0, 1.3 Hz, H-6), 6.59 (d, 2H, *J* = 8.0, 1.3 Hz, H-8); ^13^C-NMR (100 MHz, acetone-*d*_6_) δ 155.4 (C-5), 154.8 (C-4), 137.3 (C-8a), 131.2 (C-1), 129.8 (C-7), 127.6 (C-2), 119.2 (C-8), 115.8 (C-4a), 109.7 (C-6), 109.3 (C-3); HRFABMS *m/z* 317.0778 [M − H]^+^ (calcd. for C_20_H_13_O_4_, 317.0773).

### 3.4. Cell Culture and MTT Cell Viability Assay

The protective effect of the isolated compounds against cisplatin-induced renal cell damage was determined using an Ez-Cytox cell viability assay kit (DOGEN, Seoul, Korea) as reported previously [26]. LLC-PK1 cells (American Type Culture Collection, Rockville, MD, USA) were cultured in Dulbecco’s modified Eagle medium (Manassas, VA, USA) containing 10% fetal bovine serum (Invitrogen, Grand Island, NY, USA), 1% penicillin/streptomycin, and 4 mM l-glutamine. All cell culture was performed in a humidified incubator with 5% CO_2_ in air at 37 °C. Cells were seeded into 96-well culture plates (1 × 10^4^ cells/well) and treated for 2 h with the isolated compounds. Next, 25 μM cisplatin was added to each well. After incubation for a further 24 h, the cell culture media containing the compounds and/or 25 μM cisplatin was removed, and serum-free medium containing Ez-Cytox reagent was added to each well before cells were incubated for 2 h at 37 °C. Cell viability was measured by absorbance at a wavelength of 450 nm using a microplate reader (PowerWave XS; Bio-Tek Instruments, Winooski, VT, USA).

### 3.5. Image-Based Cytometric Assay

The protective effect of hypoxylonol F (**2**) against cisplatin-induced renal cell apoptosis was determined using an image-based cytometric assay following reported method [27].

LLC-PK1 cells were seeded into 6-well culture plates (4 × 10^5^ cells/well) and treated with hypoxylonol F (**2**) as described above, washed twice with cold phosphate-buffered saline, and scraped from the plates. Cells were stained with annexin V Alexa Fluor 488 and propidium iodide under dark conditions using a Tali apoptosis kit (Invitrogen, CA, USA), and analyzed using a Tali image-based cytometer (Invitrogen, CA, USA).

### 3.6. Western Blotting Analysis

The protective effect of hypoxylonol F (**2**) against cisplatin-induced renal cellular pathway was determined by Western blotting analysis [28]. LLC-PK1 cells were seeded into 6-well culture plates (4 × 10^5^ cells/well) and treated with hypoxylonol F (**2**) as described above, washed twice with cold phosphate-buffered saline. Cells were scraped from the plates and lysed in RIPA buffer (Cell Signaling Technology, Inc., Danvers, MA, USA) supplemented with 1× EDTA-free protease inhibitor cocktail and 1 mM phenylmethylsulfonyl fluoride (PMSF) according to the manufacturer’s instructions. A bicinchoninic acid protein assay was used for the quantitation of protein of samples, and 20 μg of protein were separated using 10% SDS PAGE gels for 90 min at 80 V. The proteins were transferred to PVDF transfer membranes, which were incubated with primary antibodies against phospho-p38, p38, phospho-JNK, JNK, phospho-ERK, ERK, cleaved caspase-3, and GAPDH (1:1000 dilution). Horseradish peroxidase-conjugated anti-rabbit IgG (1:2000 dilution) was used to detect the primary antibodies. Bound antibodies were visualized using ECL Advance Western Blotting Detection Reagents (GE Healthcare, Cambridge, UK). GAPDH was utilized as a loading control.

### 3.7. Statistical Analysis

The data are presented as the mean ± standard deviation (SD). Statistical significance was determined using Mann-Whitney U test. *p*-values <0.05 were considered statistically significant.

## 4. Conclusions

In this study, we describe the structural determination of three major compounds (hypoxylonol C (**1**), hypoxylonol F (**2**), and BNT (**3**)) from *H. truncatum* extract. In addition, we identified novel biological activities of hypoxylonol F (**2**) as well as the underlying molecular mechanism with respect to their renoprotective effects against cisplatin-induced nephrotoxicity in LLC-PK1 cells. In conclusion, our findings demonstrate that hypoxylonol F (**2**) suppressed apoptosis in cisplatin-induced LLC-PK1 cells by inhibiting p38, JNK, and ERK phosphorylation and caspase-3 cleavage. Taken together, these observations help to elucidate the mechanisms involved in cisplatin-induced renal damage and indicate the chemoprotective potential of hypoxylonol compounds.

## Supplementary Materials

Supplementary materials can be found at http://www.mdpi.com/1422-0067/19/4/948/s1.
