# Supplementary material for: Renoprotective Effects of Hypoxylonol C and F Isolated from Hypoxylon truncatum against Cisplatin-Induced Cytotoxicity in LLC-PK1 Cells
Source: Int J Mol Sci. 2018 Mar 22;19(4):948. doi: 10.3390/ijms19040948 (PMC5979334; doi:10.3390/ijms19040948)
Supplement: Supplementary file 1 [file ijms-19-00948-s001.pdf]

Supporting Information

Renoprotective effects of hypoxylonol C and F isolated from *Hypoxylon truncatum* against cisplatin-induced cytotoxicity in LLC-PK1 cells

Buyng Su Hwang,[1,†,‡] Dahae Lee,[2,†] Pilju Choi,[1,3,†] Kyu Sun Kim[1], Seon-Jun Choi[1], Bong Geun Song[1], Taejung Kim,[1] Ji Hoon Song,[4] Ki Sung Kang,[3,*] Jungyeob Ham[1,5*]

[1] Natural Products Research Institute, Korea Institute of Science and Technology, 679 Saimdang-ro, Gangneung 25451, Republic of Korea
[2] School of Pharmacy, Sungkyunkwan University, Suwon 16419, Republic of Korea
[3] College of Korean Medicine, Gachon University, Seongnam 13120, Republic of Korea
[4] Department of Medicine, University of Ulsan College of Medicine, Seoul 05505, Republic of Korea
[5] Division of Bio-Medical Science and Technology, University of Science and Technology, Daejeon 34113, Republic of Korea
[†] These authors contributed equally to this work
[‡] Present address: Freshwater Bioresources Utilization Bureau, Bioresources Industrialization Research Division, Nakdonggang National Institute of Biological Resources

Correspondence: kkang@gachon.ac.kr; Tel.: +82-31-750-5402 (K.S.K.) and ham0606@kist.re.kr; Tel.: +82-33-650-3502 (J.H.)

Contents

Figure S1. ^{1}H NMR spectrum of Hypoxylonol C (**1**) in acetone-d_6 2
Figure S2. ^{13}C NMR spectrum of Hypoxylonol C (**1**) in acetone-d_6 3
Figure S3. COSY NMR spectrum of Hypoxylonol C (**1**) in acetone-d_6 4
Figure S4. HSQC NMR spectrum of Hypoxylonol C (**1**) in acetone-d_6 5
Figure S5. HMBC NMR spectrum of Hypoxylonol C (**1**) in acetone-d_6 6
Figure S6. ^{1}H NMR spectrum of Hypoxylonol F (**2**) in acetone-d_6 7
Figure S7. ^{13}C NMR spectrum of Hypoxylonol F (**2**) in acetone-d_6 8
Figure S8. ^{1}H NMR spectrum of BNT (**3**) in acetone-d_6 9
Figure S9. ^{13}C NMR spectrum of BNT (**3**) in acetone-d_6 10
Figure S10-11. HRMS of Hypoxylonol C (**1**) 11-12
Figure S12-13. HRMS of Hypoxylonol F (**2**) 13-14
Figure S14-15. HRMS of BNT (**3**) 15-16

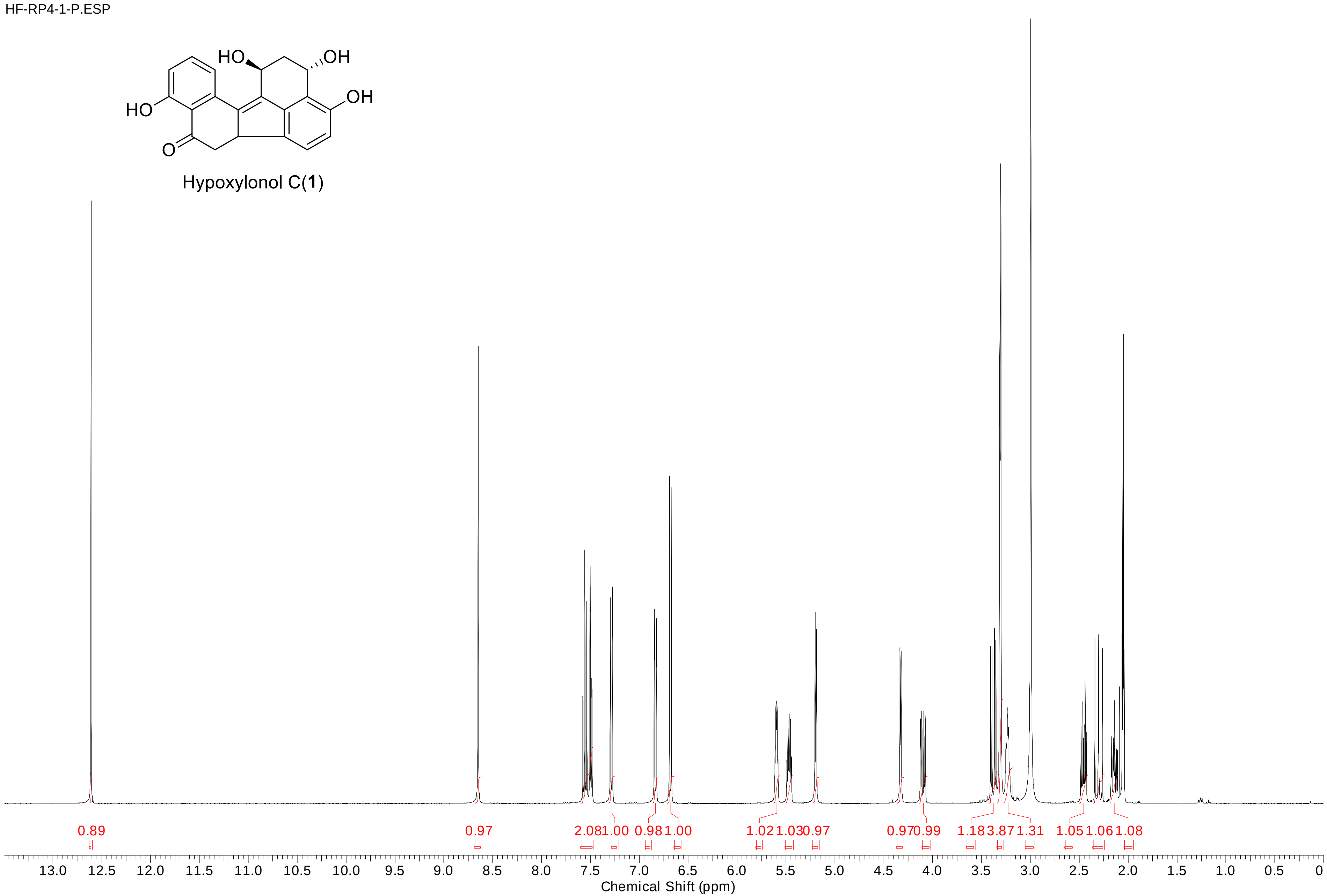

Figure S1. The ^{1}H NMR spectrum of Hypoxylonol C (1) in acetone-d_6

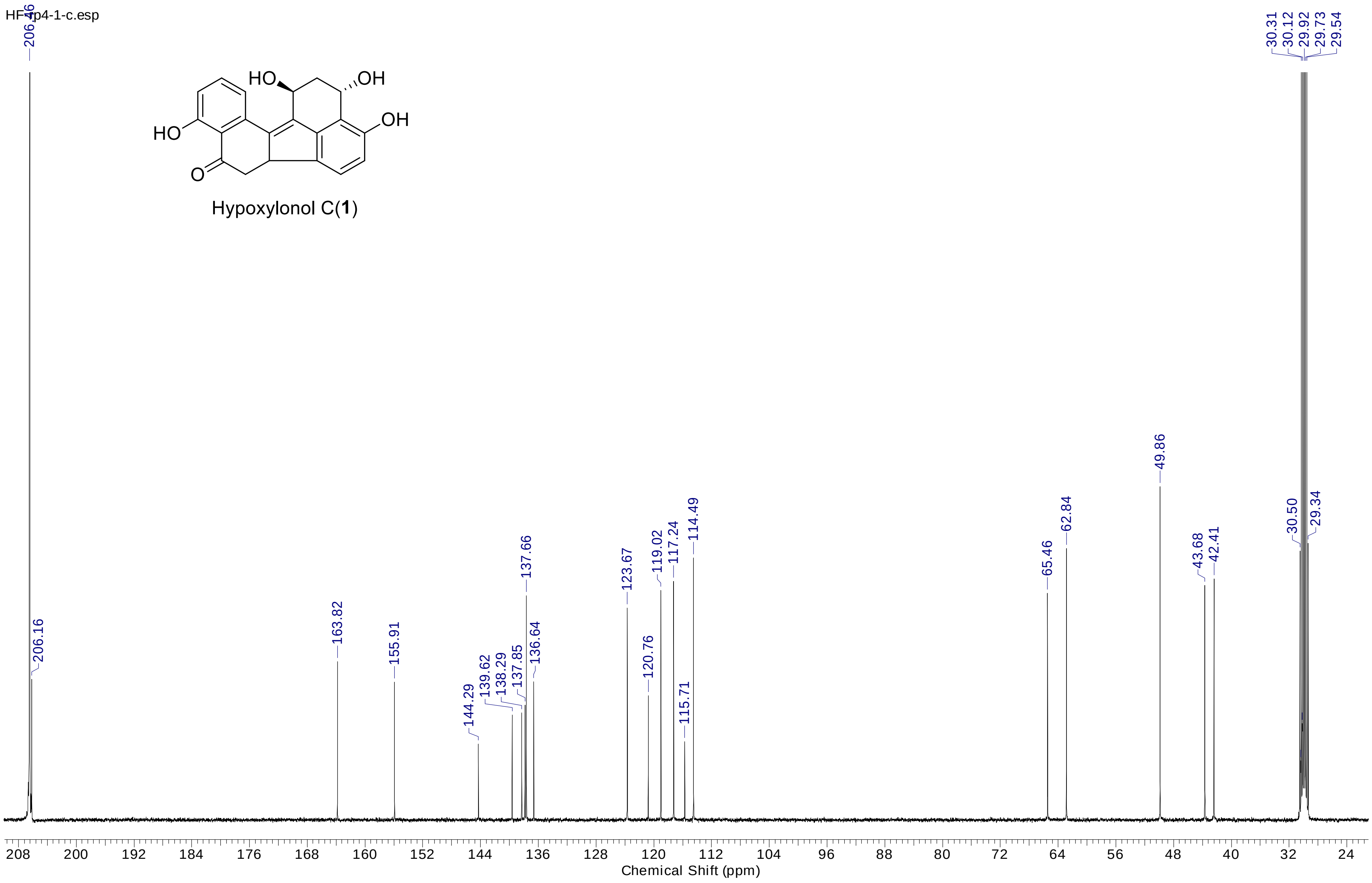

Figure S2. The ^{13}C NMR spectrum of Hypoxylonol C (1) in acetone-d_6

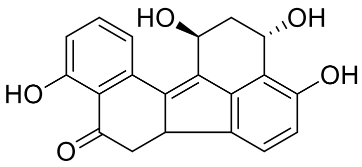

Hypoxylonol C(**1**)

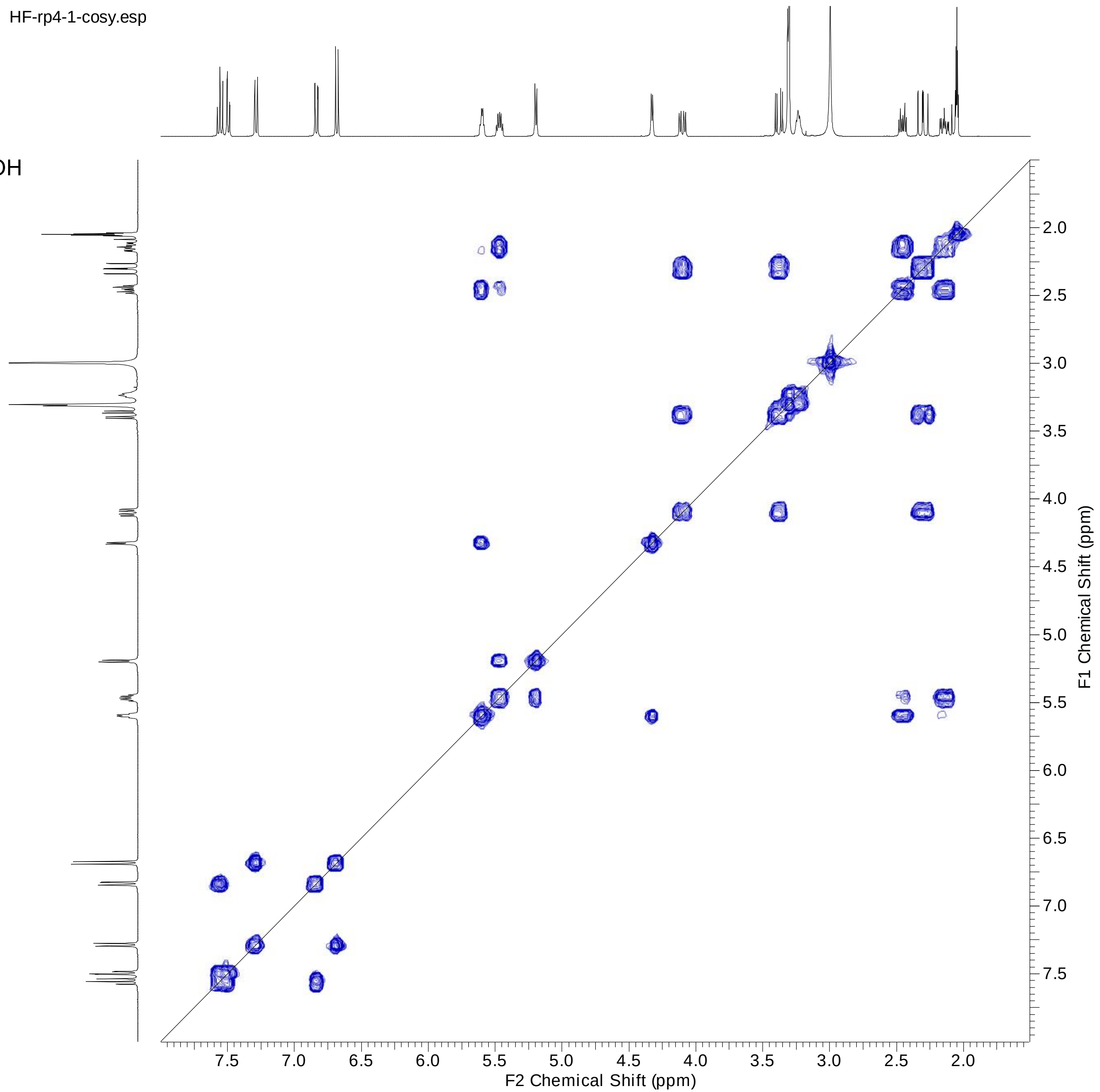

Figure S3. The COSY NMR spectrum of Hypoxylonol C (1) in acetone-d_6

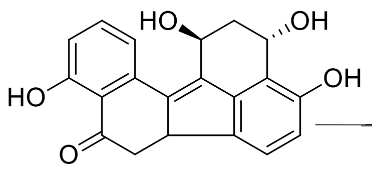

Hypoxylonol C(**1**)

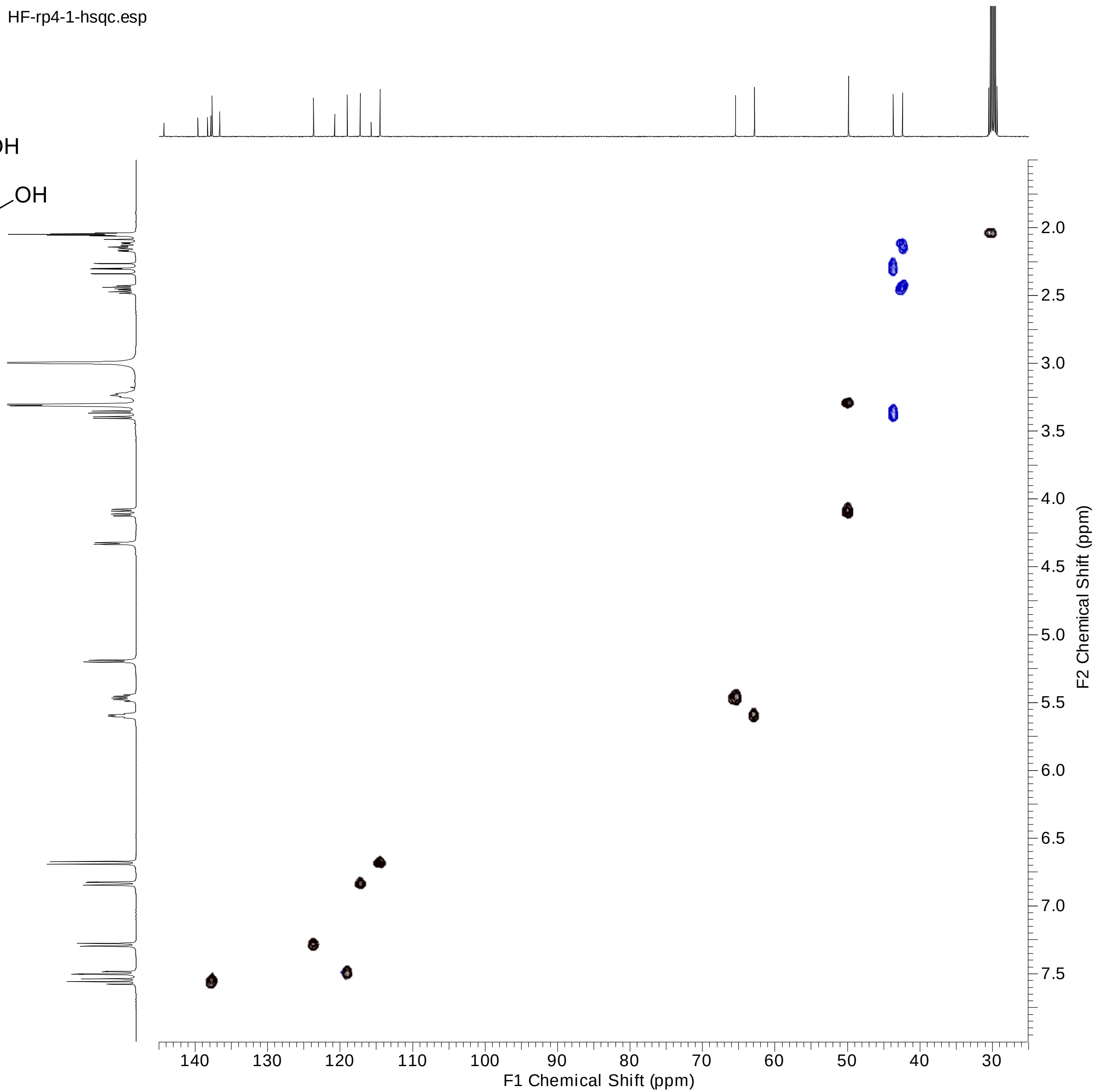

Figure S4. The HSQC NMR spectrum of Hypoxylonol C (1) in acetone-d_6 (blue colored for CH_2)

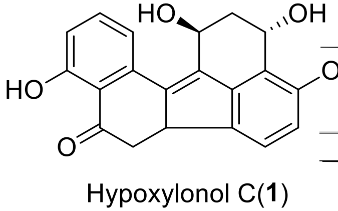

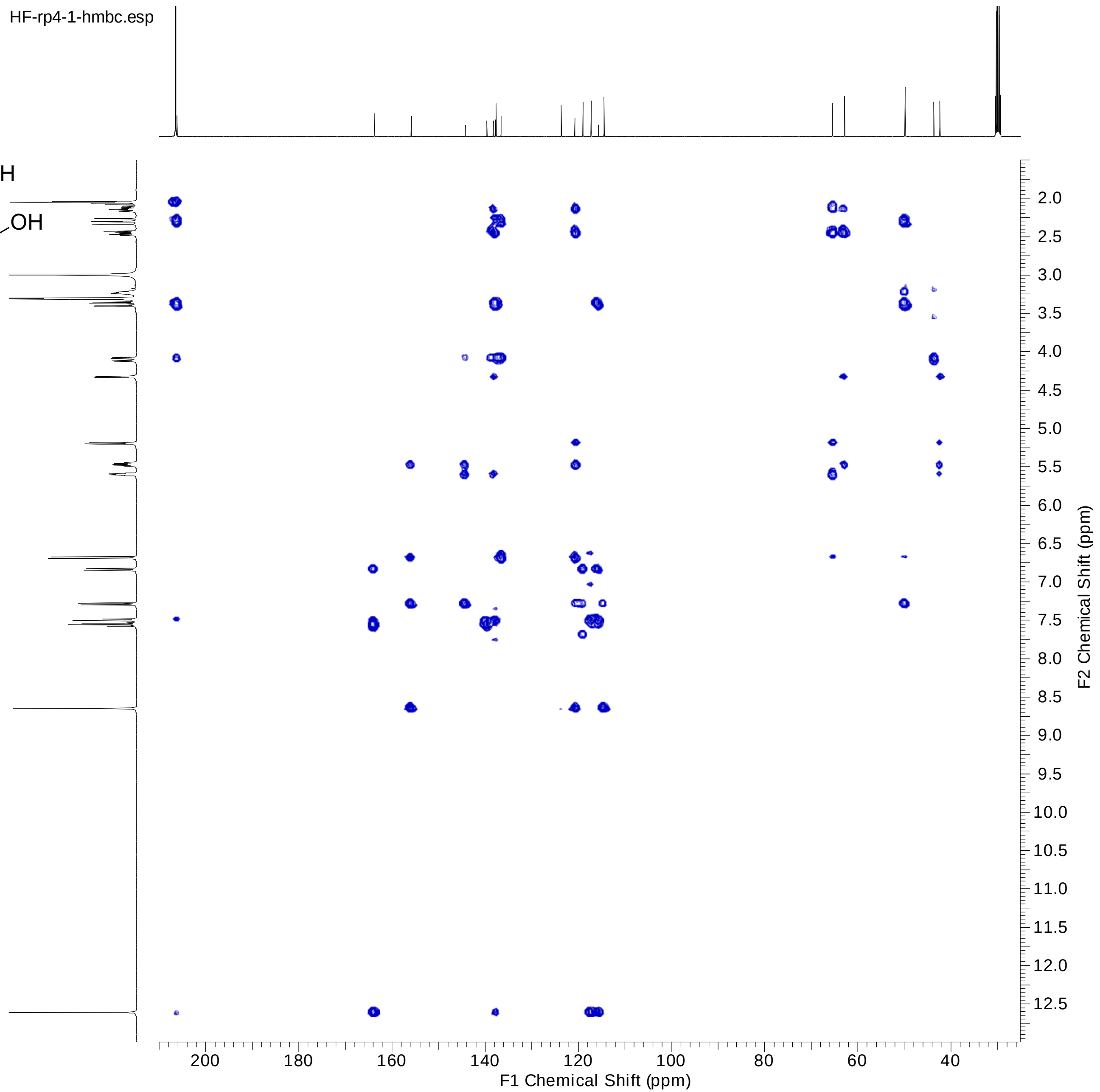

Figure S5. The HMBC NMR spectrum of Hypoxylonol C (1) in acetone-d_6

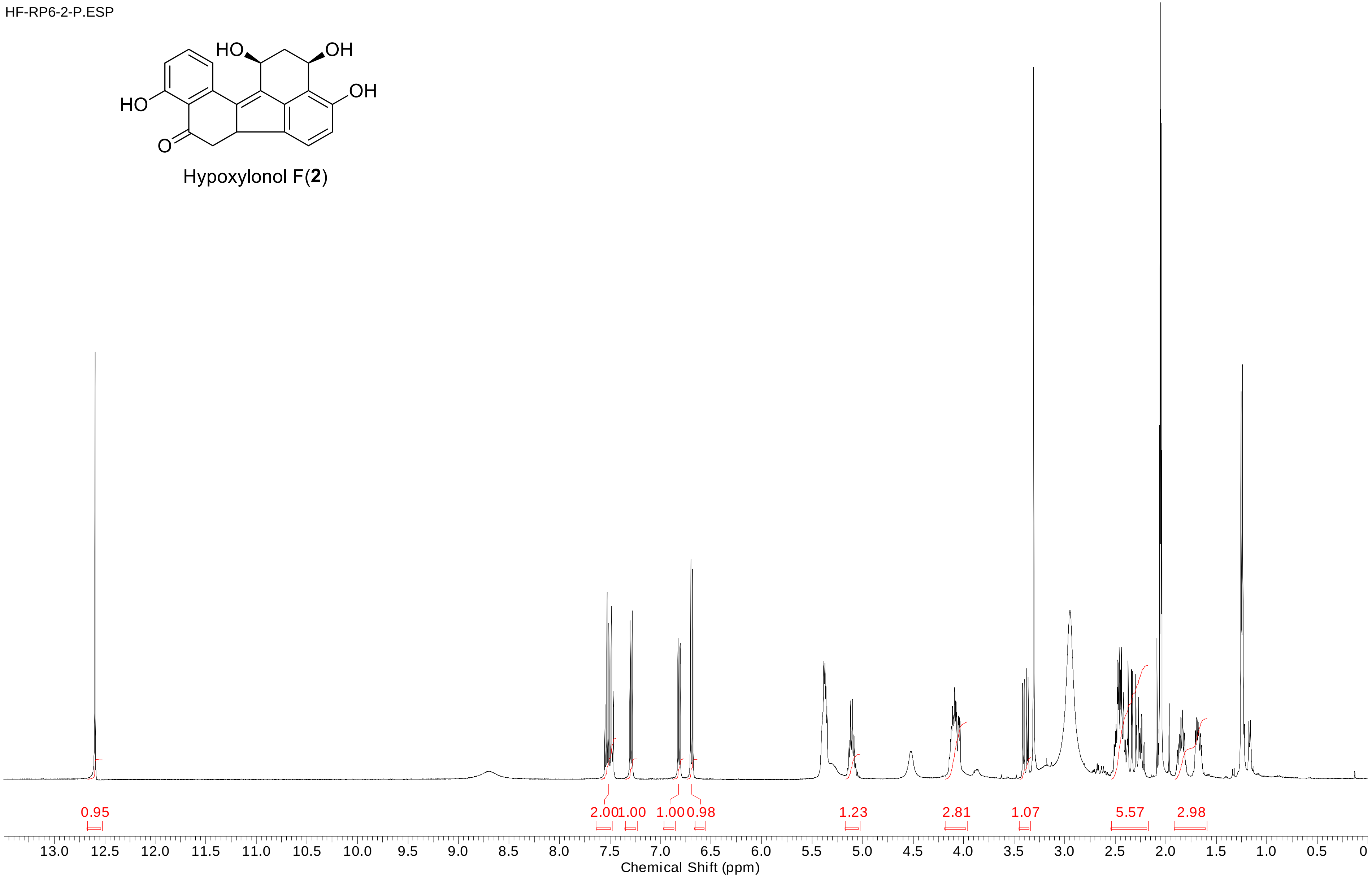

Figure S6. The ^{1}H NMR spectrum of Hypoxylonol F (2) in acetone-d_6

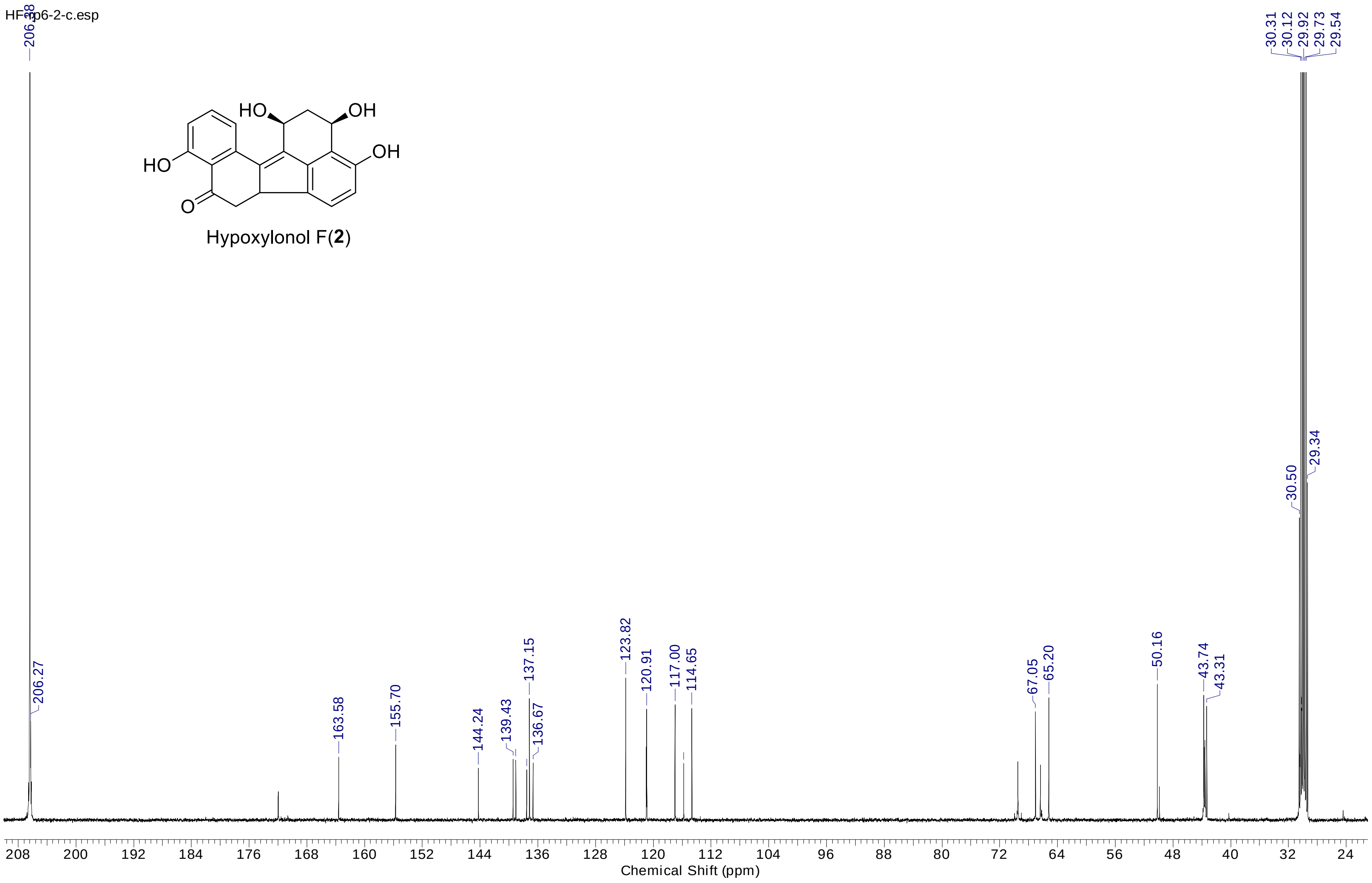

Figure S7. The ^{13}C NMR spectrum of Hypoxylonol F (2) in acetone-d_6

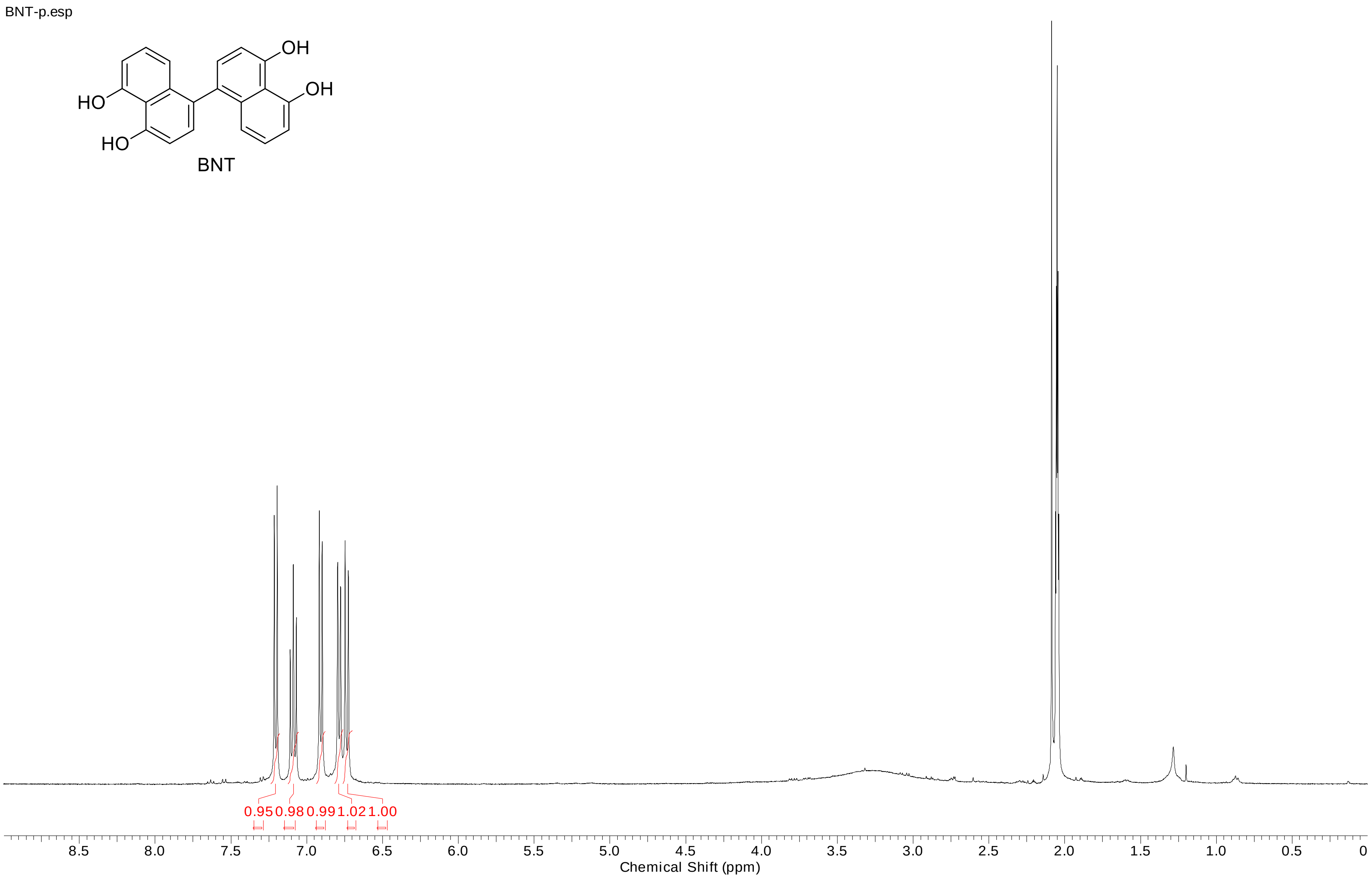

Figure S8. The ^{1}H NMR spectrum of BNT (3) in acetone-d_6

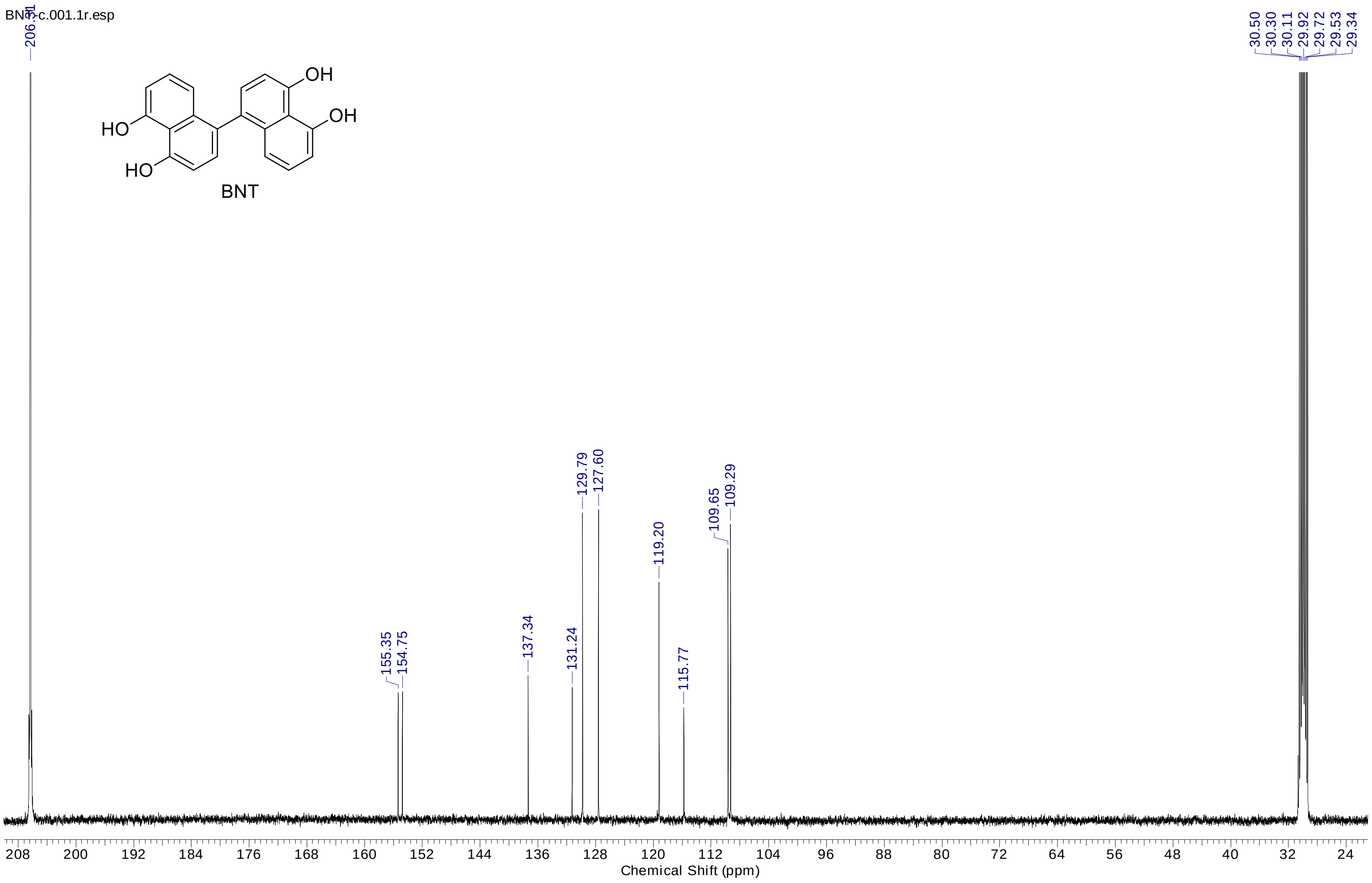

Figure S9. The ^{13}C NMR spectrum of BNT (3) in acetone-d_6

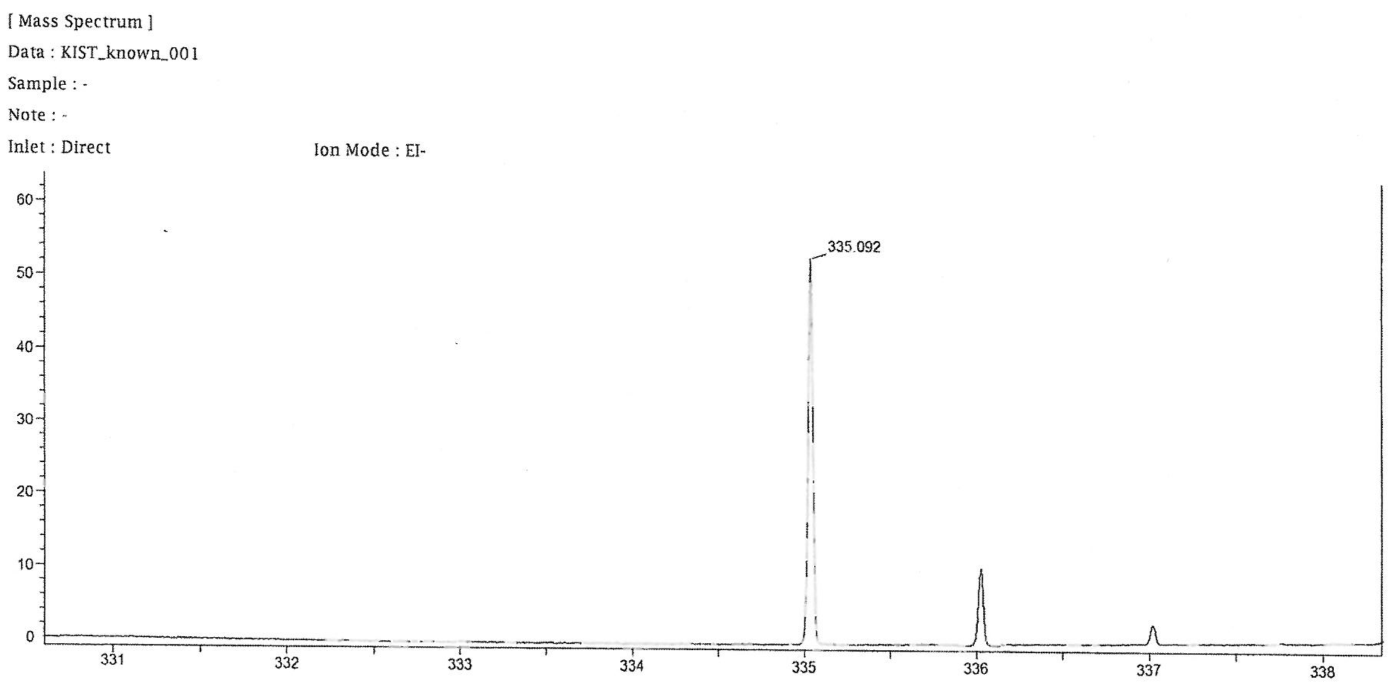

Figure S10. HRMS of Hypoxylonol C (1)

[Theoretical Ion Distribution]
Molecular Formula : C20 H15 O5 [M-H]⁻
(m/z 335.0919, MW 335.3341)
Base Peak : 335.0919, Averaged MW : 335.3145(a), 335.3316(w)

m/z	INT	
335.0919	100.0000	**
336.0942	21.4650	*************
337.0968	2.1852	**
338.0991	0.2468	
339.1104	0.0214	

Figure S11. HRMS of Hypoxylonol C (1)

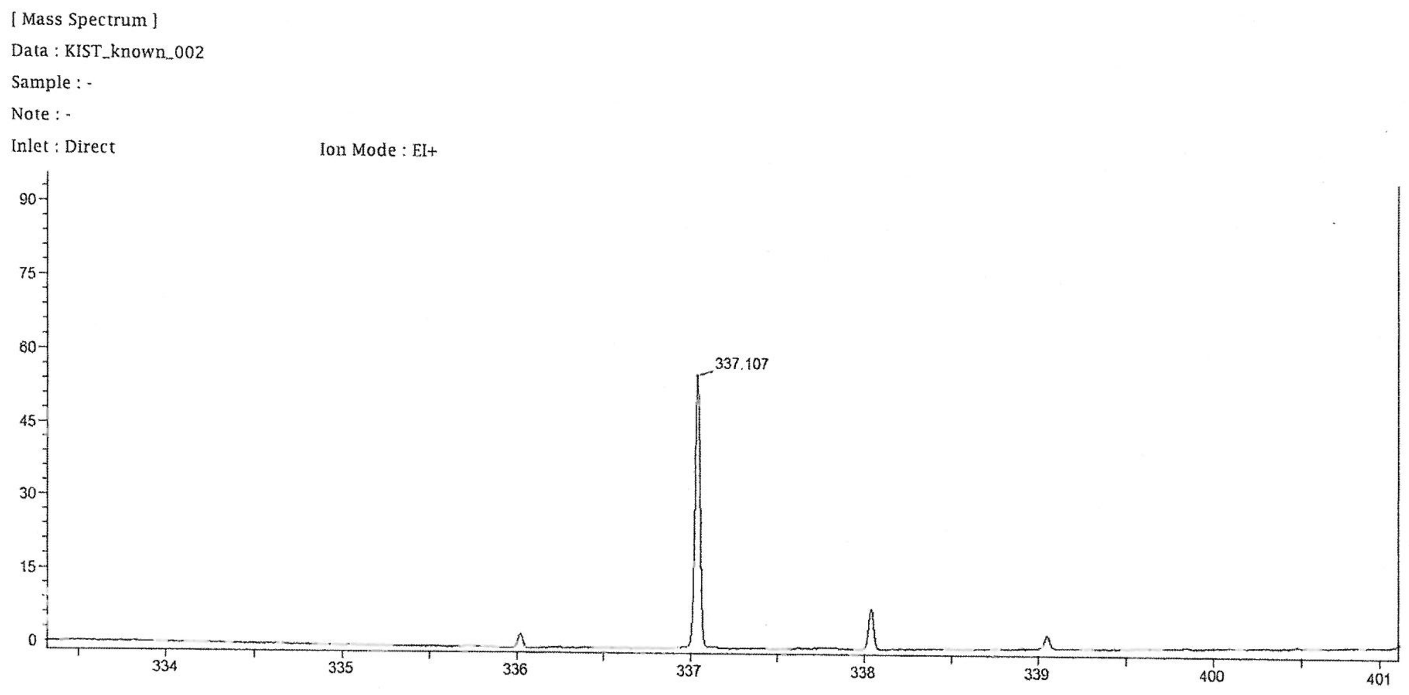

Figure S12. HRMS of Hypoxylonol F (2)

[Theoretical Ion Distribution]
Molecular Formula : C20 H17 O5 [M+H]+
(m/z 337.1074, MW 337.3511)
Base Peak : 337.1074, Averaged MW : 337.3445(a), 337.3496(w)

m/z	INT	
337.1074	100.0000	***
338.1081	21.9650	*************
339.1088	2.2852	**
400.1094	0.1975	
401.1102	0.0182	

Figure S13. HRMS of Hypoxylonol F (2)

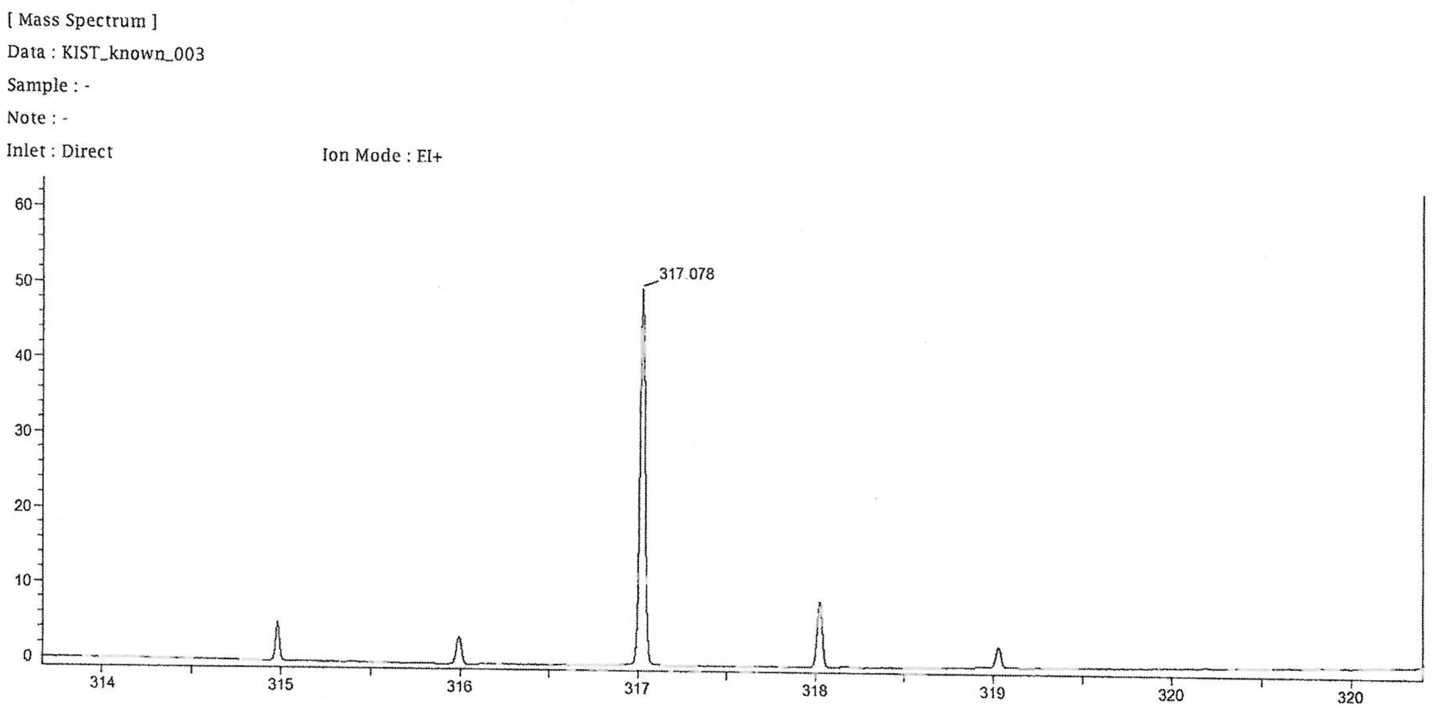

Figure S14. HRMS of BNT (3)

[Theoretical Ion Distribution]
Molecular Formula : C20 H13 O4 [M+H]+
(m/z 317.0778, MW 317.3199)
Base Peak : 317.0778, Averaged MW : 317.3045(a), 317.3156(w)

m/z	INT	
317.0778	100.0000	**
318.0792	20.8460	*************
319.0808	1.9912	**
320.0821	0.2237	
321.0834	0.0197	

Figure S15. HRMS of BNT (3)
